# Safety of transcutaneous electrical sensory stimulation of the neck in terms of vital parameters in dysphagia rehabilitation

**DOI:** 10.1038/s41598-019-49954-9

**Published:** 2019-09-17

**Authors:** Shinsuke Nagami, Keisuke Maeda, Shinya Fukunaga, Masahiro Ikeno, Yoshitaka Oku

**Affiliations:** 1 Department of Sensory Science, Faculty of Health Science and Technology, Kawasaki University of Medical Welfare, Kurashiki City, Japan; 2 Department of Physiology, Hyogo College of Medicine, Nishinomiya City, Japan; 3 Palliative Care Center, Aichi Medical University, Nagakute City, Japan; 4 Department of Nutrition and Dysphagia Rehabilitation, Tamana Regional Health Medical Center, Tamana City, Japan

**Keywords:** Ageing, Neurophysiology, Malnutrition

## Abstract

Transcutaneous electrical sensory stimulation (TESS) devices are approved for use in Japan, but their safety when used through the neck skin for dysphagia rehabilitation has not been reported. This study aimed to verify the safety of TESS use through the neck skin. Twenty patients (mean age 86.5 ± 5.1 years) with aspiration pneumonia undergoing dysphagia rehabilitation were included in this retrospective observational and matched control study. We compared vital signs in 10 subjects who underwent swallowing rehabilitation with the TESS device, and matched control patients over 7 days. The results were the following: tachycardia, 0.60 ± 1.07 vs. 0.70 ± 0.67 days; high blood pressure, 0.40 ± 0.70 vs. 0.50 ± 1.08 days; low blood pressure, 0.40 ± 0.70 vs. 0.10 ± 0.32 days; low oxygen saturation, 0.60 ± 1.58 vs. 0.50 ± 1.08 days, p = 0.870; oxygen administration, 0.80 ± 2.20 vs. 1.20 ± 2.15 days; tachypnea, 0.50 ± 0.71 vs. 0.50 ± 0.53 days; elevated body temperature, 2.00 ± 1.41 vs. 1.60 ± 1.96 days. There were no significant differences in clinical stability between the TESS and control groups of patients with aspiration pneumonia. TESS through the neck appears safe as an intervention in dysphagia rehabilitation.

## Introduction

Pneumonia is a serious problem in geriatric medicine^1^. Aspiration pneumonia among elderly people has been highlighted as a social issue in Japan, a country with a large, ageing population^2^. Aspiration pneumonia exacerbates underlying health conditions and is associated with poor outcomes^3^. In the Japanese Respiratory Society guidelines, aspiration pneumonia is defined as pneumonia caused by (or strongly suspected to be caused by) dysphagia and subsequent aspiration^4^. Because dysphagia can result in malnutrition and aspiration pneumonia, swallowing disorder is strongly linked to deteriorated quality of life. Therefore, it is important for patients with aspiration pneumonia to support oral intake as far as possible; rehabilitative interventions should be conducted accordingly^5^.

Dysphagia has various causes, including ageing, cerebrovascular diseases, head and neck tumours, and neurodegenerative diseases^6,7^. When a patient develops dysphagia, swallowing rehabilitation must be initiated promptly; however, there are no universally standard methodologies due to the wide variety of etiologies. Many approaches and procedures are being developed in different countries^6,8^.

In the United States, electrical stimulation therapy is widely used as a facilitative rehabilitation strategy to improve dysphagia. Neuromuscular electrical stimulation (NMES) to improve swallowing muscle function is effective in treating various types of dysphagia and is widely used in clinical settings^8^. Several meta-analyses have demonstrated the effectiveness of NMES against dysphagia^9^. However, a different type of electrical stimulation therapy that targets the cervical sensory nerves, unlike NMES which targets the muscles, has become popular in Japan. This therapy, known as interferential current transcutaneous electrical sensory stimulation (IFC-TESS), stimulates the afferent nerves without causing muscle contractions^10^. The animal study of Umezaki *et al*. has shown that transcutaneous IFC stimulation activates the superior laryngeal nerve (SLN) and swallowing-related neurons in the nucleus tractus solitarius (NTS)^11^. They showed that NTS neurons, which were orthodromically activated via direct electrical stimulation of the SLN, were also activated by the transcutaneous IFC stimulation. In addition, the transcutaneous IFC stimulation lowered the current threshold for eliciting swallowing reflex by direct SLN stimulation. These results indicated that IFC-TESS can activate the sensory afferent pathway (mainly the SLN) to lower the threshold for evoking the swallowing reflex. Recent studies have reported the efficacy of IFC-TESS in the rehabilitation of dysphagia^10–14^. A previous report suggested that the effects of IFC-TESS could persist as long as 15 minutes without causing any discomfort^10^.

Transcutaneous IFC stimulation activates swallowing-related neurons in the NTS and lowers the threshold for evoking the swallowing reflex^11^. However, such procedure can also activate vagal afferent fibres^15^ and may suppress inspiratory activity or lower blood pressure via the vagal reflex, which deteriorates the general condition of a patient. Such adverse effects should be prevented to safely and effectively conduct dysphagia rehabilitation. Therefore, evaluating the occurrence of adverse effects has clinical importance in refining the criteria for the application of IFC-TESS. To the best of our knowledge, the use of such method during swallowing rehabilitation does not cause any serious adverse events; however, the safety of IFC-TESS through the neck skin must be validated. Therefore, the present study aimed to evaluate the safety of TESS in clinical settings.

## Methods

### Subjects

This retrospective observational study was based on a review of the existing database and medical records of patients with aspiration pneumonia treated at the Tamana Regional Health Medical Center. This facility is a 150-bed, acute and subacute care hospital managed by the medical association of Tamana City and County. Several studies of aspiration pneumonia have been reported from the database of the hospital^5,16–18^.

The inclusion and exclusion criteria are summarised in Table 1. Patients aged 65 years or older who presented with aspiration pneumonia and underwent dysphagia rehabilitation training for >3 weeks and received TESS intervention were consecutively included in the TESS intervention group. During the study period from November 2015 to December 2016, 10 patients were included in the TESS intervention group. Among them, six participated in the previous randomised controlled trial (RCT)^13^; thus, they were randomly assigned to receive TESS. In this RCT, we evaluated the effects of TESS in dysphagia rehabilitation in terms of cough sensitivity and nutritional intake in patients with aspiration pneumonia. The other four patients who were not enrolled in the RCT were qualified for TESS therapy based on the evaluation of the speech therapists and rehabilitation doctors. No difference was observed in the background characteristics of the six patients enrolled in the RCT and the other four patients (age: 84.2 ± 5.6 vs 89.8 ± 2.9 years, p = 0.109). In addition, 10 multifactor matched patients who underwent dysphagia rehabilitation without TESS intervention were obtained from 73 consecutive cases of aspiration pneumonia during the study period. The matched factors included age, sex, severity of pneumonia, swallowing function, nutritional status and activities of daily living. The patients who wanted to withdraw from the study were excluded.

**Table 1:** Inclusion and exclusion criteria.

	Groups	
Inclusion criteria	TESS intervention	Ten patients aged 65 years or older who presented with aspiration pneumonia (AP) and underwent dysphagia rehabilitation training for >3 weeks and received TESS intervention were consecutively included in the TESS intervention group.
	Control	Ten multifactor matched patients who did not receive TESS intervention were obtained from 73 consecutive cases of AP during the study period.
Exclusion criteria	TESS intervention	Patients who wanted to withdraw from the study.
	Control	Patients who wanted to withdraw from the study.

We evaluated the stability of symptoms in patients with aspiration pneumonia. The items that should be examined were obtained from the study of Halm E. A. *et al*.^19^, which analysed the clinical stability of patients with pneumonia. The Institutional Review Board of Tamana Regional Health Medical Center approved this study (TRHMC-17-3-27), and it was conducted in compliance with the Declaration of Helsinki. Informed consent was obtained from all individual participants included in the study. Because the study was retrospective, patients who wished to be excluded from the study could do so through the opt-out method.

### Interferential current stimulation device

**Figure 1:**
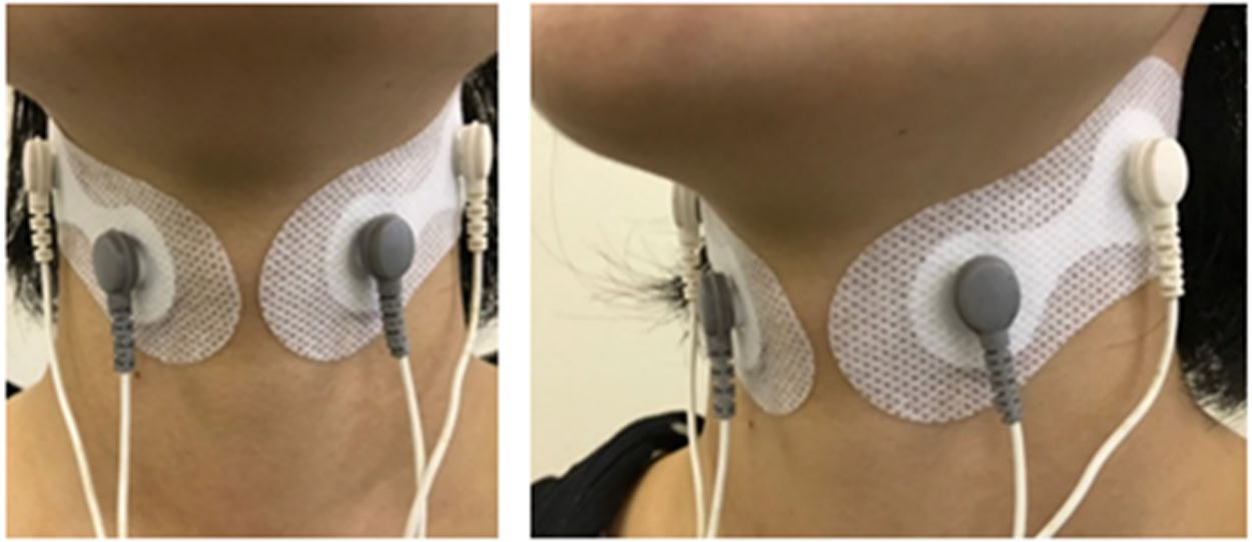
Positions of the electrodes. An interferential current of 50 beats/s was generated for sensory stimulation by the two crossing kilohertz stimulation with different frequencies (2000 and 2050 Hz).

TESS was performed with the use of an interferential current (IFC) device (Gentle Stim^®^; J Craft, Osaka, Japan). The waveform specification has been described in detail previously^10^. To obtain a 50-Hz amplitude modulation of AC cycles with 250-µs phase duration, the carrier frequency and beat frequency of the device were set at 2000 and 50 Hz, respectively. We continuously applied the IFC stimulation; thus, the duty cycle was 100%. In a preliminary study, the sensory threshold of healthy participants was found to be less than 3 mA (n = 81, mean: 1.05 mA, range: 0.18–2.18 mA; unpublished data). The sensory threshold was not measured in the present study; however, the current of the device was restricted within 3 mA by a firmware specification. Previous studies have used the device without encountering any adverse medical events^10–14^. Two electrodes generating two different frequencies (2000 and 2050 Hz) were used to stimulate the deep nerves and the surrounding tissues of the neck of patients in the TESS group. Two pair of electrodes were placed 4 cm apart on each side above and below the outer edge of the thyroid cartilage (Fig. 1) after wiping away sebum from the anterior neck with a damp towel. In all patients in the TESS intervention group, the stimulation therapy was started on the first day of dysphagia rehabilitation. Sensory stimulation was performed 5 days a week for 30 minutes at no more than 3 mA to avoid causing muscle contractions^10,12,13^. Muscle contractions were not noted via visual observation. Moreover, the current intensity of the device was limited within 3 mA by the firmware specification, which was well below the motor threshold for median nerve stimulation^20^. The patients in both groups received treatments based on the Japanese Respiratory Society guidelines for the management of community-acquired pneumonia in adults^21^ and that for the management of nursing and healthcare associated pneumonia^4^.

### Background characteristics

Data regarding the characteristics of each participant were collected from medical records. All participants were patients with aspiration pneumonia, and intervention was initiated the day after hospitalisation. Aspiration pneumonia was diagnosed when a patient fulfilled three criteria: the presence of a new gravity-dependent infiltrating shadow on chest CT imaging; the presence of two or more of the following findings—leukocytosis, fever, purulent sputum, or an elevated C-reactive protein level; and a positive result in the dysphagia screening examination or the presence of an underlying condition with a high risk of aspiration. We used the modified water swallowing test (MWST)^22,23^ to screen for dysphagia. The sensitivity and specificity of such test in differentiating between aspirators and non-aspirators were 70% and 88%, respectively^22^. The severity of pneumonia was evaluated based on age, dehydration, respiratory failure, disorientation, and low blood pressure (A-DROP severity score)^21,24^. The Functional Oral Intake Scale (FOIS)^25^ was used as an indicator of swallowing ability prior to the onset of aspiration pneumonia. The Mini Nutritional Assessment-Short Form (MNA-SF)^26^ score at admission was used as a measure of nutritional status. Additionally, the Barthel index (BI)^27^ was used as a measure of activities of daily living at admission.

### Matching criteria and outcomes

We matched patients with TESS (as cases) with those without TESS (as controls) based on age (±2 years), sex, A-DROP severity score, FOIS, MNA-SF score^26^ (±2 points), and BI^27^ (±10 points). The matching was performed one-to-one. When there were multiple candidate control patients for a single case patient, the patient with the closest date of hospitalisation to that of the case patient was chosen. To determine clinical stability, we recorded how many days out of the 7 consecutive days between the 2^nd^ and 8^th^ hospital day that the patient had a heart rate (HR) > 100 beats/min, systolic blood pressure (SBP) ≥ 160 mmHg, SBP <90 mmHg, percutaneous oxygen saturation (SpO_2_) < 90%, need for oxygen administration, respiratory rate >30/min, and body temperature >37.5 °C.

### Statistical analyses

The results associated with patient characteristics are presented as frequencies for categorical variables and means ± standard deviation for continuous variables, unless otherwise specified. The TESS and control groups were compared to examine whether clinical instability was more frequent in the TESS group. Irrespective of the presence or absence of any observed clinical exacerbation, analyses were performed using Fisher’s exact test for categorical variables and *t*-tests for continuous variables. All statistical analyses were conducted using SPSS version 21 (IBM Japan, Tokyo. Japan); a *p-*value < 0.05 was considered to indicate statistical significance.

## Results

A total of 20 patients with aspiration pneumonia (10 in the TESS group and 10 in the control group; mean age, 86.5 ± 0.5 years; 60% female) were included in the study. Matching of the 20 patients was performed based on the background characteristics shown in Table 2.

**Table 2:** Background characteristics of the patients.

Characteristic	All *(n* = 20)	Control *(n* = 10)	TESS *(n* = 10)	*P*-value
Age, yr	86.5 ± 5.1	86.5 ± 5.1	86.4 ± 5.4	0.966
Sex, *n* (%)				1.000
Female	12 (60.0)	6 (60.0)	6 (60.0)	
Male	8 (40.0)	4 (40.0)	4 (40.0)	
BMI, kg/m^2^	17.9 ± 2.5	18.4 ± 2.7	17.4 ± 2.2	0.360
MNA-SF, score	6.9 ± 2.0	7.1 ± 1.9	6.6 ± 2.1	0.586
BI, score	17.5 ± 25.8	18.5 ± 27.5	16.5 ± 25.4	0.868
FOIS, score	4.7 ± 0.8	4.7 ± 0.8	4.7 ± 0.8	1.000
Pneumonia severity, *n* (%)				1.000
Moderate	12 (60.0)	6 (60.0)	6 (60.0)	
Severe	6 (30.0)	3 (30.0)	3 (30.0)	
Extremely severe	2 (10.0)	1 (10.0)	1 (10.0)	

Abbreviations: BI, Barthel index; BMI, body mass index; FOIS, Functional Oral Intake Scale; MNA-SF, Mini Nutritional Assessment-Short Form; TESS, transcutaneous electrical sensory stimulation.

There were no significant differences between the TESS group and the control group with regard to the number of days in which abnormal parameters regarding clinical stability were observed (Table 3). All patients with TESS therapy completed planned duration and TESS intensity without being documented for the occurrence of critical symptoms.

**Table 3:** Comparison of clinical stability parameters.

Parameters	All	Control	TESS	*P*-value
	(*n* = 20)	(*n* = 10)	(*n* = 10)	
HR > 100/min, days	0.65 ± 0.88	0.60 ± 1.07	0.70 ± 0.67	0.806
SBP > 160 mmHg, days	0.45 ± 0.89	0.40 ± 0.70	0.50 ± 1.08	0.809
SBP < 90 mmHg, days	0.25 ± 0.55	0.40 ± 0.70	0.10 ± 0.32	0.232
SpO_2_ < 90%, days	0.55 ± 1.32	0.60 ± 1.58	0.50 ± 1.08	0.870
Oxygen administration, days	1.00 ± 2.13	0.80 ± 2.20	1.20 ± 2.15	0.686
RR > 30/min, days	0.50 ± 0.61	0.50 ± 0.71	0.50 ± 0.53	1.000
BT > 37.5 °C, days	1.80 ± 1.67	2.00 ± 1.41	1.60 ± 1.96	0.607

Abbreviations: BT, body temperature; HR, heart rate; RR, respiratory rate; SBP, systolic blood pressure; SpO_2_, percutaneous oxygen saturation.

## Discussion

This retrospective observational study focused on the comparisons between the TESS group and a group of patients with backgrounds similar to that of the TESS group in order to assess the medical safety of IFC-TESS. The findings of this study validate that the use of IFC-TESS through the neck skin is safe for the rehabilitation of dysphagia in patients with aspiration pneumonia. The IFC-TESS electrodes were placed on the anterior neck and generated amplitude-modulated kilohertz current stimulation in the areas of the maxillary branch of the trigeminal nerve, glossopharyngeal nerve, vagus nerve, and particularly, the SLN, which is involved in normal swallowing^28^. Various symptoms accompanying the vagal reflex must be monitored when the vagus nerve is stimulated^29,30^. The vagal reflex is characterised by blood pressure decline, bradycardia and dyspnoea. In particular, caution must be exercised when stimulation is applied to the anterior cervix because it may cause laryngeal spasm and carotid sinus reflex^31^; stimulation of the carotid sinus results in bradycardia, vasodilation and hypotension, and can cause ataxia or syncope^32^. However, in this study, the speech language therapists who performed TESS did not observe any changes in symptoms. In addition, NMES studies targeting dysphagia or facial paralysis have not reported any episodes of laryngeal spasm or carotid sinus reflex^33,34^. Although patients did not present with symptoms regarding unfavourable reactions during TESS in this study, serious autonomic responses, such as an abrupt increase in blood pressure during and immediately after therapy, which cannot be obtained from medical records, should be evaluated to conclude to a final decision regarding the safety of TESS therapy.

This study has some limitations. First, we enrolled only a small number of patients, and the observation period was short. Second, the retrospective nature of the study and the matched study design raise the possibility of bias, necessitating a large-scale prospective study to further validate the safety of TESS. Furthermore, although the most significant impact on vital signs can be measured during or immediately after TESS, the retrospective review failed to obtain the exact measurement time of every parameter. Third, as aspiration pneumonia is an infectious disease, it typically causes abnormalities of vital signs, which could have complicated the interpretation of the results. Therefore, it is imperative to validate the safety of this method in the treatment of other diseases. Finally, the occurrence of local adverse effects (such as skin irritations and allergic reactions) or general adverse effects (including discomfort) was not assessed. However, there was no documentation regarding the occurrence of vasovagal syncope or related symptoms during and immediately after TESS therapy by the attending speech therapists. Hence, further studies are warranted to overcome these limitations and assess the broader applicability of IFC-TESS.

## Conclusions

IFC-TESS through the neck skin did not cause adverse effects on vital parameters in patients with aspiration pneumonia; thus, the safety of such procedure in terms of vital parameters was validated. However, further studies of autonomic responses during and immediately after IFC-TESS and the different aspects of safety (e.g. the presence of skin irritation and anaphylaxis) must be conducted to verify the safety of IFC-TESS treatment in dysphagia rehabilitation. Further safety evaluation, which cannot be obtained from medical records, e.g. autonomic responses during and immediately after and the different aspects of safety, should be conducted to verify the safety of IFC-TESS.
